# Influence of Time of Day on Skin Microvascular Response to Post-Occlusive Reactive Hyperemia

**DOI:** 10.3390/jcm15113997

**Published:** 2026-05-22

**Authors:** Kamil Gałęziok, Krzysztof Fostiak, Artur Terbalyan, Michał Wilk, Robert Trybulski

**Affiliations:** 1 Provita Medical Centre, 44-240 Żory, Poland; rtrybulski.provita@gmail.com; 2 Department of Physical Education, Gdansk University of Physical Education and Sport, ul. Górskiego 1, 80-336 Gdansk, Poland; krzysztof.fostiak@awf.gda.pl; 3 Institute of Sport Sciences, The Jerzy Kukuczka Academy of Physical Education, 40-074 Katowice, Poland; a.terbalyan@awf.katowice.pl (A.T.); m.wilk@awf.katowice.pl (M.W.); 4 Medical Department Wojciech Korfanty, Upper Silesian Academy in Katowice, 40-664 Katowice, Poland

**Keywords:** laser Doppler flowmetry, circadian rhythm, vascular function, microcirculation

## Abstract

**Study aim:** Circadian rhythms influence numerous physiological processes, including vascular regulation and skin microcirculation. However, evidence regarding diurnal variability in post-occlusive reactive hyperemia (PORH) parameters remains limited. The aim of this study was to investigate the effect of time of day on skin microcirculatory responses assessed using laser Doppler flowmetry during a PORH test in healthy adults. **Material and methods:** Thirty healthy volunteers (15 women and 15 men) participated in the study. Skin microcirculation was assessed twice on the same day using laser Doppler flowmetry: in the morning (08:00–10:00) and in the evening (18:00–20:00). The analyzed parameters included resting flow (RF), biological zero (BZ), peak perfusion (PP), time to peak (TTP), and recovery time (TTR). A standardized occlusion protocol (100% arterial occlusion pressure for 3 min) was applied, followed by 4 min of post-occlusive monitoring. Data distribution was assessed using the Shapiro–Wilk test. Due to non-normal distribution, paired comparisons were performed using the Wilcoxon signed-rank test. Statistical significance was set at *p* < 0.05. **Results:** Resting flow was significantly higher in the evening compared with the morning (*p* = 0.038), indicating moderate diurnal variability in basal skin blood flow. No significant differences were observed for BZ, PP, TTP, or TTR (*p* > 0.05). **Conclusions:** Time of day influences resting skin blood flow but does not affect PORH parameters in healthy adults. These findings suggest that basal microvascular tone shows diurnal variability, whereas the reactive microcirculatory response remains stable. Therefore, measurement timing should be considered when interpreting resting microcirculatory assessments.

## 1. Introduction

Circadian rhythms, natural cycles that change over time, have long been recognized as profoundly affecting various physiological processes in the human body. Understanding these rhythms provides key insights into the timing and variability of biological responses in multiple systems, including hormones, metabolism, and cellular function [1]. In particular, a growing body of literature indicates that the vascular system, particularly the endothelial cells lining blood vessels, exhibits significant circadian-dependent variability in function and response. This variability is essential because it affects vascular health and the risk of various cardiovascular diseases, prompting researchers to examine the effects of temporal factors on basal skin perfusion more closely [2,3].

Laser Doppler flowmetry (LDF) is a commonly used non-invasive method for assessing skin microcirculatory blood flow. The device generates a red laser beam and penetrates deep into the tissue. Photons change their vibration frequency when they encounter red blood cells due to the Doppler effect, and then the returning light is analyzed by a photodetection system. The camera generates a voltage proportional to the speed and number of moving blood cells in the area under examination [3,4]. Combining LDF with specific reactivity tests allows us to assess the reactivity of the skin’s blood vessels. One of the reactive stimuli used in this test is occlusion, which leads to a short-term occlusion of the artery, followed by a hyperaemia response. This test is commonly known as post-occlusive reactive hyperemia (PORH). It is used to assess the vasodilatory function of the skin, characterized by markedly increased blood flow supplying a distal extremity following a period of arterial occlusion (typically 3–5 min) [5,6]. PORH refers to the increase in skin blood flow caused by brief arterial occlusion, and acute hyperaemia is mainly caused by the participation of the axon reflex of the sensory nerve and hyperpolarising factors from the endothelium (EDHF) [7,8].

Although laser Doppler flowmetry (LDF) is widely used as a non-invasive method for assessing skin microcirculation, it is important to acknowledge its methodological limitations. The technique is characterized by high spatial variability, as measurements are highly dependent on probe placement and local heterogeneity of the microvascular network. Furthermore, LDF signals are sensitive to external factors such as skin temperature, ambient conditions, and subject movement, which may influence the reproducibility and interpretation of results. In addition, variability in probe configuration and the lack of full analytical standardization may further affect measurement consistency. Therefore, strict standardization of measurement conditions is essential when using this technique [5,9,10].

Understanding the temporal dynamics of the endothelial response is crucial for both research and sports practice. Such insights can help refine risk assessment and contribute to the establishment of time-sensitive therapeutic strategies aimed at optimizing vascular health. Furthermore, healthcare professionals could use this knowledge to personalize interventions by adapting treatment regimens to individual circadian rhythms, potentially improving outcomes for patients with cardiovascular disease and recovery strategies in sports [11,12,13].

Therefore, this study aims to investigate the effect of the day on skin microcirculatory parameters assessed using laser Doppler flowmetry during a post-occlusive reactive hyperemia test in healthy individuals. Specifically, the study seeks to determine whether morning and evening measurements differ in resting skin blood flow and post-occlusive hyperemic responses. The endothelium plays a key role in regulating basal vascular tone and microvascular reactivity; however, in the skin circulation, post-occlusive hyperemia is mediated by multiple mechanisms, including neural- and endothelium-derived pathways. By comparing measurements obtained at different times of day, the present study aims to identify potential diurnal variability in skin microvascular responses under standardized conditions. It was hypothesized that skin microcirculatory parameters, including the PORH response, would differ significantly between morning and evening measurements.

## 2. Methods

### 2.1. Study Design

**Figure 1:**
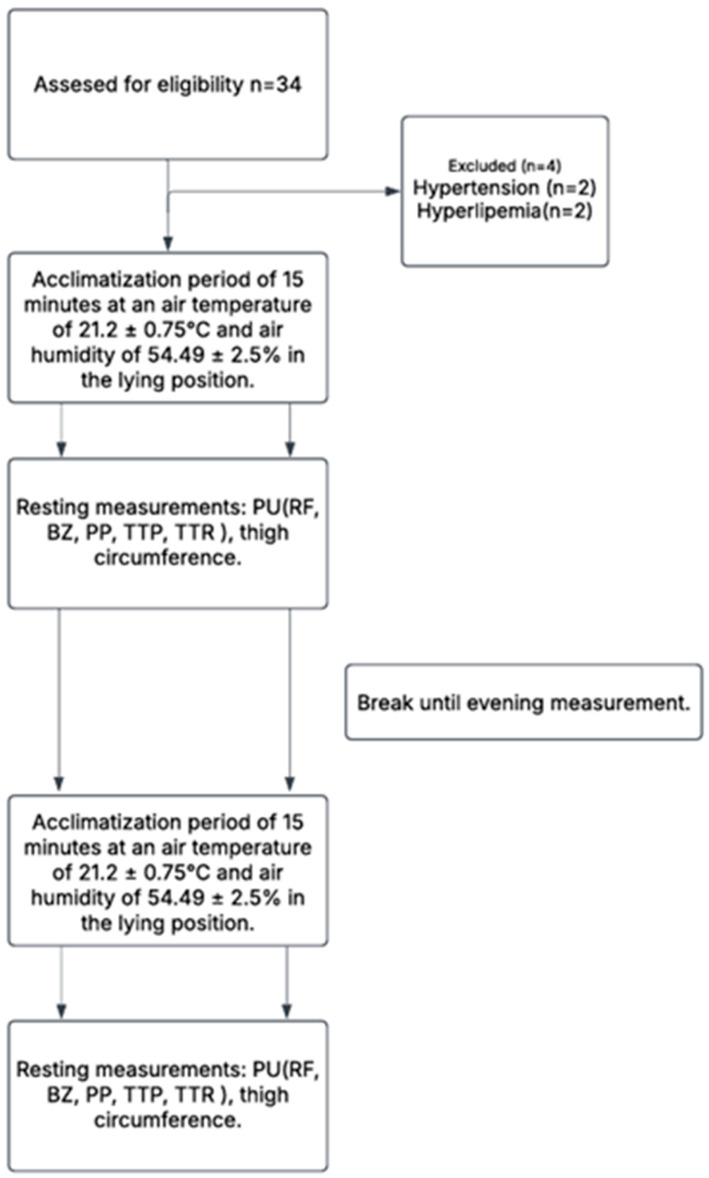
Study design. PU—perfusion units; RF—resting flow; BZ—biological zero; TTP—time to peak; TTR—recovery time; PP—peak perfusion.

This study employed a repeated-measures comparative design. The group of participants underwent a post-occlusive reaction test using laser Doppler flowmetry with Perimed device (Stockholm, Sweden, 2004) (Post-occlusive reaction hyperemia group n = 30). The study design is shown in Figure 1. Ethical approval was granted by the ethics committee of the National Council of Physiotherapists (approval number 3/03/2024, dated 27 March 2024) and the study was registered under number ISRCTN15418049. The study was in accordance with the principles set out in the Declaration of Helsinki. Participants were informed that they could withdraw from the study at any time and were also informed about possible side effects.

### 2.2. Participants

Thirty volunteers of both sexes were included in the study. Exclusion criteria were diagnosed hypertension, coronary artery disease, hyperlipemia, diabetes, and other systemic diseases that affect the microcirculation status. Smoking was also an exclusion criterion. In addition, damaged skin or unspecified skin lesions at the measurement sites and participants with a tattoo at the measurement site were also excluded because it could interfere with the LDF measurement. Exclusion was also made in case of fever, infection or at the explicit request of the participant. Written informed consent was obtained from the participants after they were informed about the study conditions. Participants were required to refrain from training for 48 h before the study, consuming ergogenic drinks and alcohol for 12 h before the study, and eating 3 h before the study. Participants could be excluded from the study at their request at any time during the study.

### 2.3. Post-Occlusive Hyperemia Reaction (PORH)

The study was conducted at the Provita Medical Center between 8:00 and 10:00 a.m. and 6:00–8:00 p.m.

The recorded air temperature was the same, 21.2 ± 0.75 °C, and air humidity, 54.49 ± 2.5%.

This study measured the following perfusion parameters [14]:

1. Resting flow (RF-[PU]), i.e., average flow before the intervention, was performed within 3 min.
2. Biological zero (BZ-[PU]), i.e., average flow during occlusion, which lasted 3 min.
3. Peak perfusion (PP-[PU]), i.e., maximum value of perfusion.
4. Time to peak (TTP-[sec]), i.e., time from opening the artery lumen to maximum flow.
5. Recovery time (TTR-[sec]), i.e., time in which perfusion returns to the resting value after the hyperemic intervention.

All measurements were performed by appropriately trained personnel.

**Figure 2:**
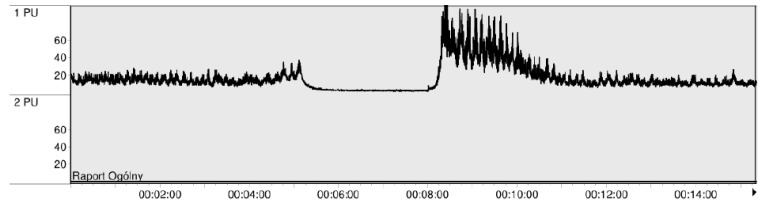
Example of LDF recording during post-occlusive reactive hyperemia (PORH).

Perimed devices (Stockholm, Sweden, 2004) were used for the measurements. The wave reflected from erythrocytes was recorded at the volume of skin tissue (1 mm^3^) and depth of 2.5 (mm) at a sampling grid of 32 [Hz]. Well-established, standardized measurements were used in this study. The assessment of tissue hyperaemia using the LDF method is highly sensitive, non-invasive and repeatable, which affects the accurate assessment of microcirculation to physical stimuli or post-stimulus responses (Figure 2) [5].

Measurements were performed on the big toe of the right foot. During the measurements, the participant was in a relaxed lying position with the leg immobilized in a neutral position in a splint. Participants underwent a 15 min acclimatization period before the study [5].

Basal perfusion measurements obtained by LDF are useful for relative within-subject comparisons but remain sensitive to environmental, autonomic, and local technical factors [5,9].

### 2.4. Protocol

Skin microcirculation was assessed using laser Doppler flowmetry (LDF). A standardized occlusion protocol was applied, consisting of arterial occlusion at 100% arterial occlusion pressure for 3 min, followed by a 4 min post-occlusive monitoring period. The following perfusion parameters were analyzed: resting flow (RF), biological zero (BZ), peak perfusion (PP), time to peak (TTP), and time to recovery (TTR). The cuff pressure after 3 min of resting flow recording was rapidly increased from 0 mmHg to 100% arterial occlusion pressure (AOP), and the occlusion was maintained for 3 min. After pressure release, microcirculatory responses were monitored using LDF, along with post-occlusive hyperemia responses for an additional 4 min [15]. The occlusion pressure was selected to ensure complete arterial occlusion in all participants. All measurements were performed consistently by the same independent physiotherapists at each designated measurement period. Depending on the duration of PORH, the entire procedure took 20 to 30 min.

**Figure 3:**
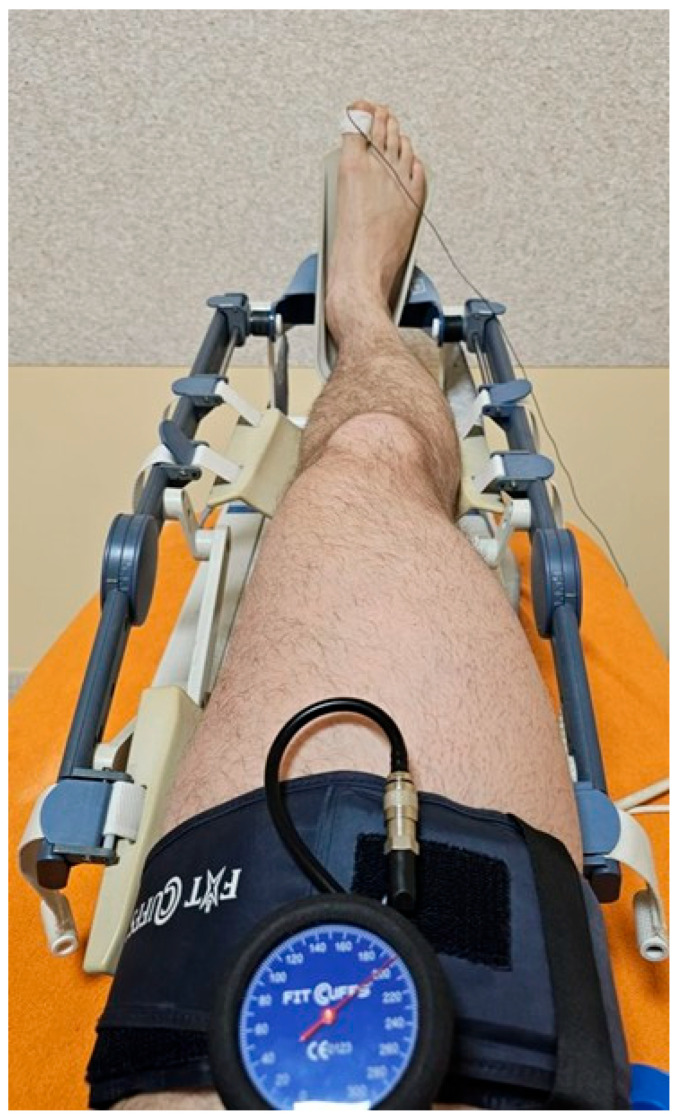
Reactive hyperemia test position.

During the measurements, the participant was in a relaxed supine position, the leg in a neutral position was stabilized in a splint to minimize artifacts during the measurement, and the patient was instructed not to move or talk during the test (Figure 3). In the same resting position, the thigh circumference was measured using a tape measure. After disinfecting the skin of the big toe, the laser Doppler flow probe was placed on the plantar side, aligning its position with the center of the nail projection, and fixed with adhesive tape, while avoiding visible veins, scars, and moles. This method is widely used to detect differences in skin perfusion and reactive hyperemia after interventions or between healthy individuals and those with cardiovascular diseases.

A 13 cm wide pneumatic cuff Riester^®^ (Jungingen, Germany) was used for occlusion, which was located at the most proximal point of the right leg. Both points were marked during the first measurement to avoid measurement differences during the second measurement caused by different device location. The measurement was performed in 3 phases.

### 2.5. Statistical Analysis

A post hoc power analysis was performed using a matched-pairs comparison framework (two-tailed, α = 0.05), corresponding to the within-subject study design. Based on the observed effect size for resting flow (estimated dz ≈ 0.40), the achieved statistical power was approximately 0.56 for a sample size of 30 participants. Although this power is below the conventional 0.80 threshold, the paired design substantially reduced inter-individual variability and increased sensitivity to time-of-day-related differences. Moreover, due to the exploratory nature of the study and the lack of robust prior data on diurnal variability of skin microcirculatory parameters assessed using post-occlusive reactive hyperemia, reliable a priori effect size estimation was not feasible at the study design stage. Importantly, the study was sufficiently sensitive to detect moderate diurnal effects, as evidenced by the statistically significant difference observed in resting flow, while non-significant findings for other parameters should be interpreted with caution [16].

All statistical analyses were conducted using Jamovi (version 2.3.21; The jamovi project, Sydney, Australia) and data visualizations were created using GraphPad Prism (version 10.1.1., GraphPad Software, Boston, MA, USA). Data are presented as means ± standard deviations (SD) for normally distributed variables, or as medians with interquartile ranges [IQR] for non-normally distributed variables. Additionally, 95% confidence intervals (95% CI) were calculated. The normality of data distributions was assessed using the Shapiro–Wilk test. For baseline comparisons, the independent Student’s *t*-test was used for normally distributed variables, with effect sizes determined using Cohen’s d. For variables that violated the normality assumption, non-parametric tests were applied. Within-subject comparisons were analyzed using the Wilcoxon signed-rank test (W). Between-subject comparisons were conducted using the Mann–Whitney U test. Where appropriate, the Bonferroni correction was applied to adjust for multiple comparisons. To evaluate the magnitude of differences the rank-biserial correlation (r_b) was calculated as the effect size. The r_b values were interpreted according to the following thresholds: |r_b| ≥ 0.10 indicating a small effect, |r_b| ≥ 0.30 a moderate effect, and |r_b| ≥ 0.50 a large effect. Statistical significance was set a priori at *p* < 0.05 for all analyses.

## 3. Results

Thirty volunteers of both sexes were included in the study according to the inclusion criteria: age 18–40 years, body mass index (BMI) 18.5–24.9. Baseline demographic and anthropometric characteristics are presented in Table 1, including a comparison between male and female participants. As expected due to sexual dimorphism, significant differences were observed in body mass (*p* < 0.001), height (*p* < 0.001), and body mass index (*p* = 0.008), with male participants presenting higher values. Conversely, age and thigh circumference did not differ significantly between the groups (*p* > 0.05).

Table 2 presents the descriptive statistics for morning and evening measurements across sexes, including mean, standard deviation (SD), median, and 95% confidence intervals (CI).

The Shapiro–Wilk test was used to assess normality. For RF, normality was not met in any condition (*p* < 0.05). BZ met the normality assumption in the morning (*p* > 0.05) but deviated from normality in the evening (*p* < 0.05). TTP met normality assumptions in morning but not evening condition (*p* > 0.05). PP satisfied normality in most conditions, except for female evening values (*p* = 0.075). TTR met normality assumptions in all cases except for morning female values (*p* = 0.025). Therefore non-parametric tests were utilized in further analyses.

**Figure 4:**
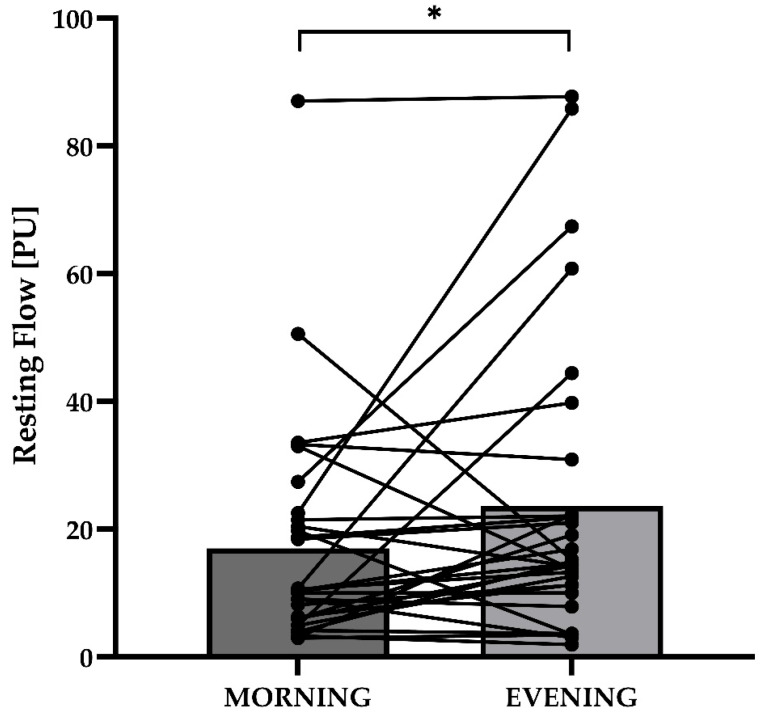
Comparison of morning and evening PORH parameters * indicates a statistically significant difference between morning and evening measurements (*p* < 0.05).

As detailed in Table 3, RF [PU] showed a significant difference with increased evening flow (W = 121^a^, *p* = 0.038) with a mean difference of −3.595 and a moderate effect size (RBC = −0.444). In contrast, the differences for BZ [PU], TTP [s], PP [PU] and TTR [s] did not reach statistical significance (*p* > 0.05). Results are presented in Figure 4 below.

Between-sex comparisons revealed no significant differences between men and women for RF, BZ, TTP, and TTR at either time point (all *p* > 0.05). For peak perfusion (PP), sex-stratified analysis revealed non-significant diurnal changes. In men, PP increased from 91.26 ± 51.81 PU in the morning to 123.50 ± 73.37 PU in the evening (*p* = 0.135, r_b = −0.45). In women, PP was 83.44 ± 53.42 PU in the morning and 79.35 ± 53.96 PU in the evening (*p* = 0.599, r_b = −0.17). The between-sex comparison for evening PP approached, but did not reach, statistical significance (*p* = 0.056, r_b = −0.413).

## 4. Discussion

The primary objective of the present study was to assess the effect of time of day on skin microvascular response during a post-occlusive reactivity test. In the analyzed material, a significant difference was observed in RF, with higher RF values recorded in the evening hours. In contrast, other (PORH) parameters, including BZ, TTP, PP and TTR, did not show significant differences between measurements performed at different times of day. Reduced resting skin perfusion observed in the morning hours has been previously reported in studies investigating circadian variability in vascular regulation. However, resting flow assessed using laser Doppler flowmetry reflects a complex interaction of multiple physiological mechanisms, including autonomic nervous system activity, vascular smooth muscle tone, local thermal regulation, hormonal fluctuations, and behavioral influences, rather than isolated endothelial function alone. Increased sympathetic nervous system activity and elevated concentrations of stress-related hormones such as cortisol and catecholamines during the early morning hours may contribute to increased vascular tone and lower basal skin perfusion. Conversely, evening measurements may reflect reduced sympathetic activity and altered vascular tone regulation, potentially contributing to higher resting perfusion values [17,18].

Importantly, despite the observed difference in RF, no significant differences were detected in PP or other PORH-derived parameters between morning and evening measurements. Therefore, the present findings should be interpreted as evidence of time-of-day-related variability in basal skin perfusion rather than definitive circadian alterations in endothelial function or reactive microvascular capacity [17,18].

In the context of PORH, although NO is a classical endothelium-dependent mediator, numerous studies indicate that the post-occlusive response in the skin microcirculation is largely governed by other mechanisms, including sensory nerve mediated axon reflexes and endothelium-derived hyperpolarizing factors EDHF. Some pharmacological studies have reported that inhibition of nitric oxide synthase does not substantially reduce the overall PORH response, which may partially explain the absence of significant differences in PORH parameters between morning and evening measurements observed in the present study [19].

This has been further confirmed by studies demonstrating that inhibition of nitric oxide synthase does not significantly reduce either the total hyperaemic response or the peak hyperaemic response following 5 or 15 min arterial occlusion. These findings may suggest that reactive hyperaemia in skin circulation is not solely dependent on NO-mediated vasodilation and that potential diurnal fluctuations in NO bioavailability may not necessarily translate into measurable differences in PORH-derived parameters under the conditions applied in the present study [20].

Additional evidence suggests that time-dependent factors may influence NO production in response to shear stress. It has been proposed that a threshold duration of increased blood flow may be required to stimulate NO synthesis, particularly during local or whole-body heating. In the study by Zhao JL et al. both separate and combined inhibition of nitric oxide synthase (NOS) and cyclooxygenase (COX) had no significant effect on any PORH variables, indicating that these pathways do not play a major role in modulating post-occlusive reactivity under the investigated conditions [21].

Although a direct relationship between the diurnal profile of prostaglandin E_2_ (PGE_2_) and vascular tone has not yet been fully characterized, available evidence suggests that temporal variability in PGE_2_ production may contribute to circadian differences in vascular tone and endothelial activity, without significantly affecting the post-occlusive hyperemic response. Studies have shown that acute inhibition of cyclooxygenase through administration of lysine acetylsalicylate (L-AS) or indomethacin does not significantly alter reactive hyperemia in the skin, indicating that prostaglandins produced via the COX pathway do not play a central role in mediating the post-occlusive response in this context [22].

These findings suggest that time-of-day-related variability in basal skin perfusion is likely multifactorial rather than solely circadian in origin. Observed differences in resting flow may reflect a combination of circadian physiology, autonomic nervous system regulation, physical fatigue, daily behavioral influences, vascular smooth muscle tone, and methodological factors such as minor probe placement variability.

Importantly, the present study did not standardize or control for chronotype, sleep duration, wake-up timing, or the interval between awakening and vascular assessment, all of which may substantially influence sympathetic activity, cortisol secretion, vascular tone, and endothelial responsiveness.

Furthermore, uncontrolled daytime factors including physical activity levels, lower-extremity fatigue, dietary intake, and hydration status may also have contributed to variability in resting perfusion measurements. Therefore, increased evening resting flow may reflect not only endogenous biological rhythms but also cumulative physiological and behavioral effects associated with daily activity patterns.

The magnitude and characteristics of the PORH response are also influenced by several protocol-related variables. It has been demonstrated that peak hyperemic response following cuff release increases with prolonged occlusion up to approximately three minutes, after which further extension of occlusion duration does not result in additional increases in response. This suggests that maximal dilation of the skin microcirculation is achieved after approximately three minutes of arterial occlusion. Similarly, increasing cuff pressure enhances the hyperemic response; however, pressures exceeding approximately 160 mmHg do not produce further significant changes. As both occlusion duration and cuff pressure were identical for morning and evening measurements in the present study, these variables did not contribute to differences in post-occlusive response between times of day [23,24].

The present findings may contribute to the understanding of time-of-day variability in basal skin perfusion under standardized conditions. However, several limitations should be acknowledged. First, the study population was relatively specific, which may limit the generalizability of the findings to individuals with different fitness levels, age groups, or chronic health conditions. Additionally, laser Doppler flowmetry (LDF) measurements reflect relative rather than absolute perfusion and remain sensitive to probe placement, local tissue heterogeneity, and environmental conditions, which may influence measurement reproducibility.

Furthermore, an important methodological nuance emerged regarding peak perfusion (PP). As noted in our results, men exhibited a substantial morning-to-evening increase in PP accompanied by a large effect size (r_b = −0.45), whereas women displayed a slight decrease. Because these sex-specific responses were in opposite directions, they effectively canceled each other out in the pooled cohort analysis, resulting in a statistically insignificant diurnal difference for the general sample (Table 3). Although the within-group changes did not reach strict statistical significance—likely constrained by the subgroup sample sizes (n = 15)—this observation strongly points to a potential sexual dimorphism in maximal vasodilatory capacity across the day. Future studies with larger cohorts should specifically investigate these sex-dependent diurnal microvascular responses to prevent masking effects in gender-aggregated analyses.

Furthermore, segmented area-under-the-curve (AUC) analyses for basal, occlusion, and reperfusion phases were not performed, which may have limited the sensitivity of the study to detect more subtle changes in PORH dynamics.

Future studies would benefit from incorporating AUC-based analyses, phase-specific perfusion quantification, and standardized control of behavioral and sleep-related factors to better isolate endogenous circadian influences on skin microvascular regulation. Additional investigations using varied occlusion protocols, gradual arterial lumen closure, and assessments across different body regions may further enhance understanding of PORH physiology. Moreover, exploration of underlying mechanisms, including potential diurnal variability in nitric oxide and prostaglandin activity during occlusive procedures, could provide a more comprehensive understanding of microvascular regulation.

The relatively small sample size should also be acknowledged as a limitation of this study, as it may have limited the statistical power to detect subtle diurnal differences in PORH-related parameters. Consequently, non-significant findings should be interpreted cautiously.

Despite these limitations, the present findings suggest that while resting skin perfusion may exhibit time-of-day-related variability, post-occlusive reactive hyperemic responses remain relatively stable between morning and evening measurements under the tested conditions.

## 5. Conclusions

The present study demonstrates a significant effect of time of day on resting skin blood flow, reflected by higher flow response values in the evening compared to morning measurements. In contrast, no significant diurnal differences were observed in PORH parameters, indicating preserved basal skin perfusion independent of time of day. These findings suggest that basal skin perfusion demonstrates time-of-day–related variability, although this may reflect a complex interaction of circadian, behavioral, autonomic, and environmental influences. Further studies are warranted to elucidate the specific pathways underlying diurnal variations in vascular function and their clinical relevance.

## Figures and Tables

**Table 1:** Demographic characteristics and anthropometric data of the participants by sex and pooled.

Variable	Men (n = 15)	Women (n = 15)	Test Statistic	*p*	Effect Size	Pooled (n = 30)
Age [years]	27.9 ± 5.4	26.4 ± 5.5	*U* = 134	0.380	*r*_b = −0.19	27.1 ± 5.4
Body mass [kg]	80.2 ± 11.3	60.4 ± 9.2	*t*(28) = 5.26	<0.001 *	*d* = 1.92	70.3 ± 14.3
Height [cm]	180.3 ± 7.3	165.9 ± 6.1	*t*(28) = 5.87	<0.001 *	*d* = 2.14	173.1 ± 9.9
BMI [kg/m^2^]	24.6 ± 3.1	21.8 ± 2.3	*t*(28) = 2.84	0.008 *	*d* = 1.04	23.2 ± 3.0
Thigh circumference [cm]	57.5 ± 4.8	53.9 ± 7.1	*t*(28) = 1.59	0.122	*d* = 0.58	55.7 ± 6.2

Values are mean ± SD. SD—standard deviation; *t*—Student’s *t*-test statistic; U—Mann–Whitney U; d—Cohen’s d; r_b—rank-biserial correlation. * *p* < 0.05.

**Table 2:** Basic descriptive statistics of variables by sex and pooled.

Variable	Time	Group	Mean ± SD	Median [IQR]	95% CI	Shapiro–Wilk *p*
RF [PU]	Morning	Men	17.30 ± 21.21	10.00 [4.94, 19.75]	5.56–29.05	<0.001
		Women	16.72 ± 13.87	10.39 [6.32, 27.42]	9.04–24.39	0.023
		Pooled	17.01 ± 17.61	10.36 [5.99, 21.22]	10.71–23.31	<0.001
	Evening	Men	26.47 ± 27.20	14.98 [10.00, 22.25]	11.41–41.53	0.001
		Women	20.79 ± 19.06	14.58 [11.24, 22.07]	10.24–31.35	0.001
		Pooled	23.63 ± 23.26	14.78 [11.25, 22.20]	15.31–31.95	<0.001
BZ [PU]	Morning	Men	2.24 ± 0.87	2.27 [1.57, 2.71]	1.75–2.72	0.228
		Women	2.39 ± 0.71	2.46 [1.99, 2.81]	2.00–2.78	0.748
		Pooled	2.31 ± 0.78	2.44 [1.66, 2.70]	2.03–2.60	0.208
	Evening	Men	3.33 ± 4.10	2.37 [1.30, 3.03]	1.07–5.60	<0.001
		Women	2.74 ± 1.55	2.52 [2.16, 2.55]	1.88–3.60	0.002
		Pooled	3.04 ± 3.06	2.50 [1.52, 3.16]	1.94–4.13	<0.001
TTP [s]	Morning	Men	39.13 ± 14.88	37.00 [29.50, 44.00]	30.89–47.37	0.120
		Women	43.40 ± 25.15	30.00 [24.00, 59.00]	29.48–57.32	0.061
		Pooled	41.27 ± 20.42	36.00 [24.00, 58.25]	33.96–48.57	0.012
	Evening	Men	48.73 ± 24.98	42.00 [29.50, 70.00]	34.89–62.56	0.085
		Women	43.60 ± 20.05	36.00 [27.00, 56.50]	32.50–54.70	0.059
		Pooled	46.16 ± 22.41	39.00 [29.25, 62.25]	38.15–54.18	0.010
PP [PU]	Morning	Men	91.26 ± 51.81	79.20 [57.80, 121.00]	62.57–119.95	0.274
		Women	83.44 ± 53.42	85.40 [33.20, 112.50]	53.86–113.02	0.506
		Pooled	87.35 ± 51.86	80.00 [54.55, 122.80]	68.79–105.91	0.332
	Evening	Men	123.50 ± 73.37	103.50 [68.90, 169.70]	82.87–164.13	0.189
		Women	79.35 ± 53.96	65.70 [29.45, 142.40]	49.47–109.24	0.075
		Pooled	101.43 ± 67.14	77.05 [51.25, 142.48]	77.40–125.45	0.027
TTR [s]	Morning	Men	101.40 ± 38.89	89.00 [77.00, 120.00]	79.86–122.94	0.874
		Women	99.85 ± 61.43	90.00 [75.00, 117.00]	65.83–133.87	0.025
		Pooled	100.63 ± 50.53	89.50 [77.25, 120.00]	82.55–118.71	0.009
	Evening	Men	117.80 ± 39.39	109.00 [87.00, 137.00]	95.99–139.61	0.929
		Women	110.33 ± 51.78	112.00 [60.00, 135.00]	81.66–139.01	0.362
		Pooled	114.07 ± 45.36	112.00 [84.75, 136.50]	97.83–130.30	0.436

SD—standard deviation; IQR—interquartile range; CI—confidence interval; *p*—test probability (Shapiro–Wilk test); RF—resting flow; BZ—biological zero; TTP—time to peak; PP—peak perfusion; TTR—recovery time; PU—perfusion units.

**Table 3:** Wilcoxon signed-rank test results.

Variable 1	Variable 2	W-Statistic	*p*	Mean Difference	SE Difference	Effect Size (RBS)
MORNING RF [PU]	EVENING RF [PU]	121	0.038 *	−3.595	3.600	−0.444
MORNING BZ [PU]	EVENING BZ [PU]	162	0.230	−0.220	0.473	−0.257
MORNING TTP [s]	EVENING TTP [s]	186	0.344	−3.500	5.791	−0.200
MORNING PP [PU]	EVENING PP [PU]	165	0.171	−9.400	11.399	−0.290
MORNING TTR [s]	EVENING TTR [s]	162	0.147	−17.500	8.689	−0.305

RBS—rank biserial correlation, *p*—test probability, *—statistical significance, RF—resting flow, BZ—biological zero, TTP—time to peak, TTR—recovery time, PU—perfusion units.

## Data Availability

The original contributions presented in this study are included in the article. Further inquiries can be directed to the corresponding author.

## Abbreviations

The following abbreviations are used in this manuscript:

AOP	arterial occlusion pressure
BMI	body mass index
BZ	biological zero
CI	confidence interval
COX	cyclooxygenase
EDHF	endothelium-derived hyperpolarizing factor
L-AS	lysine acetylsalicylate
LDF	laser Doppler flowmetry
NO	nitric oxide
NOS	nitric oxide synthase
PGE_2_	prostaglandin E_2_
PORH	post-occlusive reactive hyperaemia
PP	peak perfusion
PU	perfusion units
RBC	rank biserial correlation
RF	resting flow
SD	standard deviation
SE	standard error
TTP	time to peak
TTR	time to recovery
